# Affective state of people suffering from long covid and associated factors. Cross-sectional descriptive study

**DOI:** 10.1192/j.eurpsy.2023.1642

**Published:** 2023-07-19

**Authors:** B. Oliván-Blázquez, M. Samper-Pardo, S. León-Herrera, A. Aguilar-Latorre, F. López-Mendez, R. Magallón-Botaya

**Affiliations:** 1 University of Zaragoza; 2 Institute for Health Research Aragón, Zaragoza, Spain

## Abstract

**Introduction:**

The “Post-COVID Syndrome” affects approximately 10% of people who have been infected with Covid-19. These people have a physical and mental impact.

**Objectives:**

The objective of this study is to analyze factors related to poorer mental health in these patients from primary health care.

**Methods:**

Cross-sectional study. The study population was post-COVID-19 patients aged 18 years or older and treated by Primary Health Care (PHC). The main variable was Affective state through the Hospital Anxiety and Depression Scale (HADS) questionnaire. The rest of the variables were: Socio-demographic variables, number of residual symptoms, cognitive using the Montreal Cognitive Assessment (MoCA), physical functioning variable will be measured by Sit to Stand Test and Sleep quality through the Insomnia Severity Index (ISI). A bivariate analysis and also a lineal multivariate model were developed. Ethics approval was granted by the Clinical Research Ethics Committee of Aragón (PI21/139 and PI21/454).

**Results:**

A total of 100 individuals participated, of whom, 80 were women and 20 were men. The median scores in HADS was 16 and the interquartile range was 12. Multilevel analysis shows that better physical functioning (sit to stand test) and worse sleep quality (Insomnia severity index) are predictors of worse affective state. The models explain 36.5% of the HADS variance.

**Conclusions:**

It is relevant to take account these variables in the treatment of the affective state of patients with long covid.

**Disclosure of Interest:**

None Declared

